# The U5 snRNA Internal Loop 1 Is a Platform for Brr2, Snu114 and Prp8 Protein Binding During U5 snRNP Assembly

**DOI:** 10.1002/jcb.24625

**Published:** 2013-07-16

**Authors:** Verity Nancollis, Jayalath PD Ruckshanthi, Lily Novak Frazer, Raymond T O'Keefe

**Affiliations:** Faculty of Life Sciences, The University of ManchesterMichael Smith Building, Oxford Road, Manchester, M13 9PT, United Kingdom

**Keywords:** PRE-mRNA SPLICING, SPLICEOSOME, U5 snRNP, Brr2, Snu114, Prp8, *SACCHAROMYCES CEREVISIAE*

## Abstract

The U5 small nuclear ribonucleoprotein particle (snRNP) forms the heart of the spliceosome which is required for intron removal from pre-mRNA. The proteins Prp8, Snu114 and Brr2 all assemble with the U5 small nuclear RNA (snRNA) to produce the U5 snRNP. Successful assembly of the U5 snRNP, then incorporation of this snRNP into the U4/U6.U5 tri-snRNP and the spliceosome, is essential for producing an active spliceosome. We have investigated the requirements for Prp8, Snu114 and Brr2 association with the U5 snRNA to form the U5 snRNP in yeast. Mutations were constructed in the highly conserved loop 1 and internal loop 1 (IL1) of the U5 snRNA and their function assessed in vivo. The influence of these U5 mutations on association of Prp8, Snu114 and Brr2 with the U5 snRNA were then determined. U5 snRNA loop 1 and both sides of IL1 in U5 were important for association of Prp8, Snu114 and Brr2 with the U5 snRNA. Mutations in the 3′ side of U5 IL1 resulted in the greatest reduction of Prp8, Snu114 and Brr2 association with the U5 snRNA. Genetic screening of *brr2* and U5 snRNA mutants revealed synthetic lethal interactions between alleles in Brr2 and the 3′ side of U5 snRNA IL1 which reflects reduced association between Brr2 and U5 IL1. We propose that the U5 snRNA IL1 is a platform for protein binding and is required for Prp8, Brr2 and Snu114 association with the U5 snRNA to form the U5 snRNP. J. Cell. Biochem. 114: 2770–2784, 2013. © 2013 The Authors. Journal of Cellular Biochemistry Published by Wiley Periodicals Inc.

Accurate removal of intron regions from pre-messenger RNA (pre-mRNA) is catalysed by the spliceosome, a large RNA-protein complex composed of five small nuclear RNAs (snRNAs) and numerous proteins [Wahl et al., 2009]. Intron removal by the spliceosome is essential for production of mature messenger RNA with the correct reading frame for protein production by the ribosome. Additionally, in higher eukaryotes alternative splicing of introns provides increased diversity of protein products from a single gene [Chen and Manley, 2009].

The core functional units of the spliceosome are the five small nuclear ribonucleoprotein particles (snRNPs) that each contain an snRNA (U1, U2, U4, U5 or U6), Sm or LSm proteins and proteins unique to each snRNP [Wahl et al., 2009]. The snRNPs interact with the pre-mRNA to allow precise recognition and removal of intron regions. Assembly of snRNPs with the pre-mRNA begins with the binding of the U1 snRNP to the 5′ splice site. The U2 snRNP then binds to the branch site before the pre-assembled U4/U6.U5 tri-snRNP arrives to form the complete spliceosome. In addition to the stepwise pathway of spliceosome assembly, there is also evidence for pre-assembled tetra-snRNP (U2.U4/U6.U5) and penta-snRNP (U1.U2.U4/U6.U5) particles associating with the pre-mRNA to form the spliceosome [Gottschalk et al., 1999; Stevens et al., 2002]. The fully assembled spliceosome is not competent to catalyse intron removal until the NineTeen Complex (NTC) of proteins associates with the spliceosome [Hogg et al., 2010] and the spliceosome is remodelled by eight ATPases and one GTPase to form active conformations required for the two steps of intron removal [Smith et al., 2008].

The U5 snRNP is the most highly conserved snRNP, being conserved from yeast to humans [Newman, 1997]. Moreover, the U5 snRNP is the only common snRNP found in the major U2-dependent and minor U12-dependent spliceosomes [Patel and Steitz, 2003]. The U5 snRNA contains a conserved structure that includes the essential loop 1 sequence [Frank et al., 1994]. The U5 snRNA loop 1 interacts directly with the 5′ exon before the first step of splicing and with the 5′ and 3′ exons following the first step of splicing [Newman and Norman, [Bibr b29],[Bibr b30]; Wyatt et al., 1992; Sontheimer and Steitz, 1993; Newman et al., 1995; O'Keefe et al., 1996; O'Keefe and Newman, 1998; Alvi et al., 2001; McGrail et al., 2006; McGrail and O'Keefe, 2008]. These U5–exon interactions are essential for tethering and aligning the exons for ligation during the second step of splicing [O'Keefe and Newman, 1998]. The U5 snRNA stem 1 and internal loop 1 (IL1) are also highly conserved between yeast and humans, both in size and in structure [Frank et al., 1994].

The U5 snRNP contains proteins that are essential for remodelling the spliceosome during splicing and may contribute to the active site of the spliceosome. The protein composition of the U5 snRNP is also highly conserved from yeast to humans. The common U5 snRNP proteins between yeast and humans are Prp8, Snu114, Brr2, Prp28, Snu40/52K and Dib1 [Stevens et al., 2001]. During activation of the spliceosome for catalysis, it is only Prp8, Snu114 and Brr2 that remain associated with the U5 snRNA with the core Sm proteins [Fabrizio et al., 2009]. Prp8 is a 280 KDa protein with no obvious homology to other proteins [Grainger and Beggs, 2005]. It is a component of the U5 snRNP and is also part of the U4/U6.U5 tri-snRNP. Prp8 forms a salt-resistant complex with the ATPase Brr2 and the GTPase Snu114 without the U5 snRNA suggesting that they may interact with the U5 snRNA as a complex [Achsel et al., 1998]. Prp8 also interacts with other proteins of the spliceosome, the snRNAs and extensively with the pre-mRNA. It has been shown that Prp8 crosslinks directly to the pre-mRNA 5′ splice site, the branch site and the 3′ splice site as well as U5 and U6 snRNAs localising it to the heart of the spliceosome [Grainger and Beggs, 2005]. In yeast, Prp8 makes extensive direct contacts with the U5 snRNA including the highly conserved U5 loop 1 and IL1 [Dix et al., 1998]. Prp8 is believed to be the master regulator of the splicing cycle by regulating the spliceosomal DExD/H-box RNA dependent ATPases, specifically Brr2 [Collins and Guthrie, 2000; Grainger and Beggs, 2005]. Consistent with this regulatory role it has been shown that the C-terminus of Prp8 activates Brr2 helicase function and inhibits Brr2's U4/U6-dependent ATPase activity in vitro [Maeder et al., 2009]. Recent structural studies of the Prp8 C-terminus have identified an RNase H-like domain within Prp8 and it has been proposed that this RNase H domain may form the active site of the spliceosome [Abelson, 2008]. The ATPase Brr2 is required for unwinding U4/U6 base-pairing before the first step of splicing and for unwinding U2/U6 base-pairing after the second step of splicing [Hahn and Beggs, 2010]. These conformational changes catalysed by Brr2 are essential for the progression of the spliceosome cycle. Recent structural analysis of Brr2 has revealed that part of the Sec63 like cassettes of the Brr2 helicase domains resemble the DNA helicase Hel308, hinting at an RNA unwinding action of Brr2 similar to that of the DNA unwinding by helicase Hel308 [Pena et al., 2009; Zhang et al., 2009]. The activity of Brr2 is regulated by the guanine nucleotide state of Snu114 [Small et al., 2006]. Snu114 is the only GTPase associated with the spliceosome [Frazer et al., 2008]. Snu114 displays extensive genetic interactions with the proteins and snRNAs of the spliceosome [Brenner and Guthrie, 2005; Frazer et al., 2009]. Overall, this triumvirate of Prp8, Snu114 and Brr2, together with the U5 snRNA, are essential for spliceosome function.

Proper assembly of the U5 snRNP is required for formation of the U4/U6.U5 tri-snRNP and its incorporation into the spliceosome. Little is known about the regions of the U5 snRNA required for association of Prp8, Snu114 and Brr2 with the U5 snRNP, and if the requirements for their association with U5 are different for each protein. Defining the requirements for association of Prp8, Snu114 and Brr2 with the U5 snRNP will provide information on U5 snRNP assembly, whether Prp8, Snu114 or Brr2 bind U5 snRNA independently and if different regions of the U5 snRNA are required for association of each protein. To identify the regions of the U5 snRNA important for association of Prp8, Snu114 and Brr2 we have constructed a series of U5 snRNA mutants within the highly conserved loop 1 and IL1. The association of Prp8, Snu114 and Brr2 with these U5 snRNA mutants was then assessed by immunoprecipitation of the proteins from yeast whole cell extracts. The U5 snRNA IL1 was found to be the most important region for association of Prp8, Snu114 and Brr2 with the U5 snRNA. Genetic analysis also identified the U5 snRNA IL1 as being important for Brr2 function. Overall, the U5 snRNA IL1 appears to be critical for association of the U5 snRNP proteins Prp8, Snu114 and Brr2 with the U5 snRNA to form the U5 snRNP.

## MATERIALS AND METHODS

### Yeast Strains

C-terminally TAP-tagged *SNU114* and *BRR2* strains were produced by transformation of yeast strain BJ2168 [Jones, 1991] with a PCR amplified cassette from plasmid pYM13 [Janke et al., 2004] for chromosomal integration by homologous recombination. BJ2168 was used in extract preparation for Prp8 immunoprecipitation. BJ2168 or TAP-tagged *SNU114* and *BRR2* strains were transformed with plasmid pROK4 (U5 + ins) or U5 mutants in pROK4 (U5 + ins) to produce extracts for immunoprecipitations. Viability of U5 mutants in plasmid pROK4 (U5 + ins) and m571 were tested in strain YROK2 [O'Keefe, 2002].

### Preparation of Yeast Whole Cell Extracts and Isolation of RNA From Extracts

Yeast whole cell extracts were produced by the liquid nitrogen breakage method [Ansari and Schwer, 1995; Alvi et al., 2001]. For RNA isolation yeast extract (25 µl) was diluted with 125 µl water and 50 µl proteinase K stop mix (1 mg/ml proteinase K, 50 mM EDTA, 1% SDS). Reactions were incubated at 37°C for 15 min. An equal volume of citrate buffered (pH 5.3) phenol–chloroform–isoamyl alcohol (PCA) was added and reactions were extracted four times. Aqueous phase was brought to 0.3 M sodium acetate and RNA precipitated with 2.5 volumes of ethanol. Precipitated RNA was resuspended in 20 µl water.

### Immunoprecipitation of TAP-Tagged Proteins and Associated RNA From Yeast Extracts

Rabbit IgG agarose beads (Sigma—50 µl) were washed three times in IPP150 (10 mM Tris–Cl pH 8, 150 mM sodium chloride, 0.1% IGEPAL). The final wash was removed and 100 µl yeast whole cell extract containing TAP-tagged protein was added with 300 µl of IPP150, then incubated at 4°C for 2.5 h. Beads were washed four times with 1 ml IPP150, the last wash was removed then 400 µl splicing diluent (300 mM sodium acetate pH 5.3, 1 mM EDTA, 0.1% SDS, 25 µg/ml tRNA) and 400 µl PCA were added. Samples were extracted four times. The final supernatant was transferred to a new tube, 2 µg tRNA and 2.5 volumes of ethanol were added to precipitate the RNA. Precipitated RNA was resuspended in water.

### Immunoprecipitation of Prp8 and Associated RNA From Yeast Extracts Using Prp8 Antibodies

Protein A Sepharose CL-4B beads (GE Healthcare—40 mg) were washed four times with water then resuspended in 600 µl IPP150 without IGEPAL (10 mM Tris–Cl pH 8, 150 mM sodium chloride). Prp8 antibody (R1703, supplied by J. Beggs) was added to 70 µl beads and incubated at 23°C for 2 h. Beads were washed three times with IPP150 without IGEPAL. The final wash was removed and 150 µl yeast extract and 150 µl IPP150 without IGEPAL were added followed by incubation on a roller at 4°C for 2 h. Beads were washed four times with IPP150 without IGEPAL. The last wash was removed then 400 µl splicing diluent and 400 µl PCA were added. Samples were extracted four times. The final supernatant was transferred to a new tube, 2 µg tRNA and 2.5 volumes of ethanol were added to precipitate the RNA. Precipitated RNA was resuspended in water.

### Primer Extension Analysis

All RNA from TAP tag or antibody immunoprecipitation reactions was used in a single primer extension reaction. Only 0.5 µl of RNA purified from whole cell yeast extracts was used in each primer extension. For primer extension RNA was hybridised with radiolabelled primer U5RT (Table S1) in 1× RT buffer (Roche). Reactions were heated to 90°C and cooled to 41°C. Reactions were increased to 20 µl with the addition of 1× RT buffer (Roche), 7.35 µl dNTP/DTT mix (1 mM each dNTP, 10 mM DTT), 10 units RNAsin (Promega), 3.3 units AMV reverse transcriptase (Roche). Reactions were incubated at 41°C for 30 min. Splicing diluent (180 µl) was added then 200 µl Tris (pH 8) buffered PCA was added for extraction of primer extension products. The aqueous phase was transferred to a new tube and primer extension products precipitated with the addition of 2.5 volumes of 100% ethanol and incubation at −20°C. Primer extension products were resuspended in 1 µl water and 4 µl formamide loading dye and separated on a 40 cm 6% Sequagel at 32 W for 2 h. Fixed and dried gels were either exposed to autoradiography film (Fuji) or exposed to phosphorimaging screen (Fuji, BAS cassette 2040) for quantification with a BioRad Molecular Imager FX. The wild-type U5 snRNA band was used as loading control for any variability in immunoprecipitation, and data was normalised using this wild-type U5 snRNA band. Background readings were subtracted from all values. All data collected for U5 + ins mutants were normalised, where U5 + ins was equal to 1 and the reading for the lane containing only wild-type U5 (not U5 + ins) was equal to 0. Experiments were repeated in triplicate, except in the case of the experiment investigating the effects of deletions in the 3′ side of U5 snRNA IL1 on associations of Snu114, which was only repeated twice. Error bars in Figures 5 show the mean ± standard deviation between replicates.

### Construction of *brr2* and U5 snRNA Mutants

The *brr2* mutants were constructed by oligomutagenesis of plasmid pRS413-Brr2. The U5 snRNA mutants were constructed in plasmid pROK4 which contained a 20 nucleotide sequence insertion (AGAAGTATGCAAAGCATGCA) in the *SNR7* gene corresponding to U5 snRNA stem 2 between positions U121 and C122. Plasmid pROK4 was constructed by in vitro mutagenesis of plasmid m571 [O'Keefe et al., 1996]. The U5 snRNA mutants to test for genetic interactions with *brr2* mutants were constructed in plasmid pRS415-U5. All mutagenesis primers are listed in Table S1. The resulting plasmids were sequenced to identify the correct mutation.

### Synthetic Genetic Analysis

A haploid double knockout strain for *BRR2* and *SNR7* (U5 snRNA) (MATa; ura3-52; his3Δ200; leu2Δ0; YER172C::kanMX4; SNR7::hphNT1; pRS416-Brr2-U5) was transformed with mutated pRS413-Brr2 and pRS415-U5, then transformants selected on synthetic defined (SD) medium (SD-Ura-His-Leu) (BIO 101 Systems). Transformants were then tested for synthetic lethality by plasmid shuffle on 5-FOA. Synthetic lethality was scored as the lack of growth and synthetic sickness by minimal growth after 3 days at 25°C.

### Analysis of Brr2/U5 Association of Selected Synthetic Lethal Interactions

TAP-tagged *BRR2* was PCR amplified from genomic DNA prepared from the BJ2168 *BRR2*-TAP strain with Phusion DNA polymerase (New England Biolabs) and primers Brr2FG and Brr2BG (Table S1). The PCR product was cloned into pRS415 to produce pRS415-BRR2TAP and confirmed by sequencing. The R295I and R1107A mutations were then introduced into pRS415-BRR2TAP and the U5 ΔC112G113 mutation was introduced into pROK4 (U5 + ins) by mutagenesis and all confirmed by sequencing. Plasmids were then transformed into BJ2168 to produce strains with pRS415-BRR2TAP-R295I or pRS415-BRR2TAP-R1107A and pROK4 (U5 + ins) or pROK4 (U5 + ins) ΔC112G113. Extracts produced from these strains were then used for immunoprecipitation with IPP150 without IGEPAL then primer extension as described above.

## RESULTS

### Analysis of U5 snRNA Mutants In Vivo

**Fig. 1:**
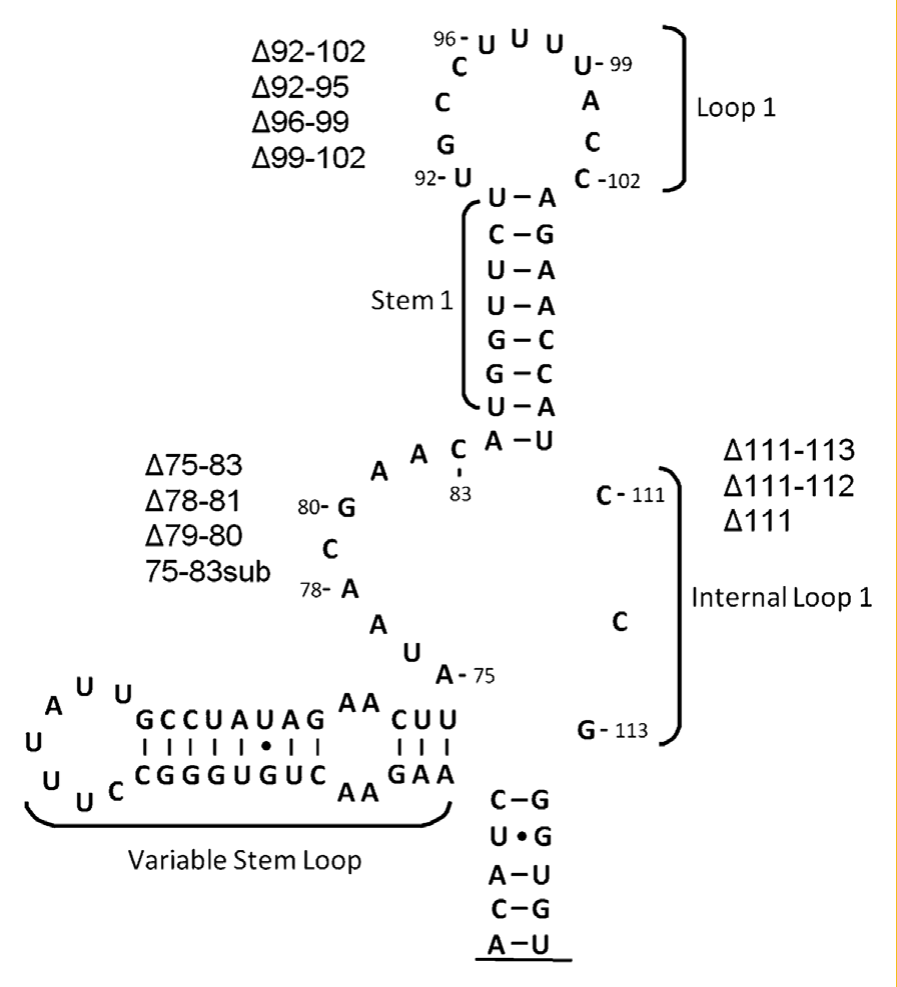
U5 snRNA mutants designed to investigate association of Brr2, Snu114 and Prp8. Diagram of the *Saccharomyces cerevisiae* U5 snRNA. Three regions of U5 snRNA in plasmid pROK4 (U5 + ins) were mutated to investigate the influence of mutation on the association of U5 snRNP proteins. The three regions chosen for investigation were U5 snRNA Loop 1 and the 3′ and 5′ side of internal loop 1 (IL1). The mutations constructed are listed on the sides of the U5 snRNA near the mutated region. Only nucleotides 37–118 of the 214 nucleotide full length U5 snRNA are shown.

Prp8, Snu114 and Brr2 are known to associate with the U5 snRNA to form the U5 snRNP [Achsel et al., 1998]. However, little is known of the U5 snRNA requirements for Prp8, Snu114 and Brr2 association with the U5 snRNA. To define the regions of U5 snRNA required for the association of Prp8, Snu114 and Brr2 with U5 snRNA in yeast, U5 snRNA mutants were constructed for use in immunoprecipitations. A reduction in association of a U5 snRNA mutant with a U5 snRNP protein, compared with wild-type U5 snRNA, would suggest that the mutated region is involved in the association of that U5 snRNP protein with the U5 snRNA. Three regions of the U5 snRNA were chosen for mutagenesis to allow investigations into the U5 requirements for Prp8, Snu114 and Brr2 association with U5 (Fig. 1). The first region chosen for mutagenesis was the 5′ side of U5 snRNA IL1. The 5′ side of IL1 was investigated because it is essential for U5 snRNA function, and both Snu114 and Prp8 are known to crosslink to position C79 in the IL1 region [Frank et al., 1994; Dix et al., 1998]. The 3′ side of U5 snRNA IL1 was also chosen for analysis because it is conserved from humans to yeast, is required for U5 snRNA function and Prp8 crosslinks to the IL1 region [Frank et al., 1994; Dix et al., 1998]. In human U5 snRNA, both sides of IL1 (called IL2 in humans) are necessary for efficient expression of U5 snRNA, U5 snRNP formation and spliceosome assembly [Hinz et al., 1996]. The third region chosen for analysis was loop 1 of the U5 snRNA. U5 loop 1 was chosen for its high conservation, essential function, vital role in aligning exons for ligation, and as the site of Prp8 crosslinking [Newman and Norman, [Bibr b29],[Bibr b30]; Frank et al., 1994; O'Keefe et al., 1996; Dix et al., 1998; O'Keefe and Newman, 1998]. Studies on the human U5 snRNA have shown that loop 1 is involved in human Prp8 binding [Hinz et al., 1996; Urlaub et al., 2000].

**Fig. 2:**
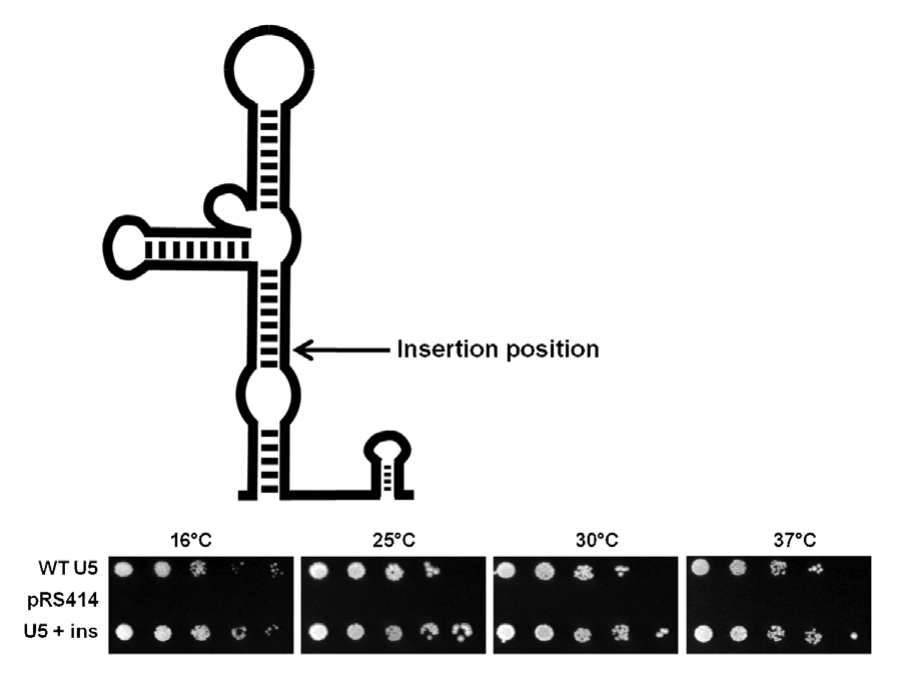
In vivo analysis of U5 + ins plasmid used for U5 snRNA mutant production. A 20 nucleotide sequence was inserted into stem 2 of U5 snRNA (U5 + ins) between nucleotides U121 and C122, allowing differentiation in size between mutant and wild-type U5 snRNAs (top). Plasmid shuffle reveals that the 20 nucleotide insert in U5 snRNA (U5 + ins) does not influence the viability of cells as the sole source of U5 snRNA. One in five serial dilution was performed from a starting OD_600_ of 1. Each dilution was spotted onto 5-FOA containing plates. Each plate included a positive control strain containing wild-type U5 in pRS414, and a negative control strain containing pRS414. Spotted plates were incubated at 16, 25, 30 and 37°C.

Four different mutations were constructed in the 5′ side of U5 snRNA IL1 (nucleotides 75–83). The first mutation was deletion of the whole 5′ side of IL1, nucleotides 75–83 (Δ75–83). The 5′ side of U5 snRNA IL1 was also subjected to two smaller deletions, with nucleotides 78–81 (Δ78–81) and 79–80 (Δ79–80) being deleted. A final mutant was constructed in which nucleotides 75–83 in the 5′ side of IL1 were substituted with the complement of the wild-type sequence (75–83 sub) (Fig. 1). Four deletions were made in loop 1 of the U5 snRNA, the first being deletion of the entire loop 1, nucleotides 92–102 (Δ92–102). Loop 1 was also deleted in three smaller sections, nucleotides 92–95 (Δ92–95), 96–99 (Δ96–99) and 99–102 (Δ99–102) (Fig. 1). Finally, three mutants were constructed containing deletions in the 3′ side of U5 snRNA IL1. The first mutation made in the 3′ side of IL1 was deletion of nucleotides 111–113 (Δ111–113), the second being deletion of nucleotides 111 and 112 (Δ111–112) and the final mutation being a single nucleotide deletion of nucleotide 111 (Δ111) (Fig. 1). As U5 snRNA mutants are often lethal, U5 mutants were constructed in the pROK4 plasmid (referred to as U5 + ins). This pRS314 plasmid contains the U5 snRNA gene with a 20 nucleotide insertion (U5 + ins) between positions U121 and C122 in stem 2 of the U5 snRNA (Fig. 2). Constructing U5 snRNA mutants in the U5 + ins plasmid enables differentiation of the wild-type and mutant U5 snRNAs by size and also allows analysis of lethal mutations as wild-type U5 is still present.

U5 snRNA mutants constructed in the U5 + ins plasmid were tested for viability using a plasmid shuffle assay in a yeast strain in which the gene encoding wild-type U5 snRNA, *SNR7*, was deleted. As U5 snRNA is essential, the U5 snRNA deletion was complemented with wild-type *SNR7* present on a *CEN*/*URA3* plasmid. This strain was transformed with U5 snRNA mutants (in U5 + ins) and colonies were transferred to 5-fluoro-orotic acid (5-FOA) containing media, to select against the *URA3* plasmid containing wild-type U5 snRNA. Growth on 5-FOA containing media resulted in the mutant U5 snRNA being the sole source of U5 snRNA, and following growth of yeast at 16, 25, 30 and 37°C, it can be determined if the U5 mutant is viable, lethal, cold or temperature sensitive. The viability of U5 + ins was tested to confirm that the presence of the unique 20 nucleotide insert did not affect viability compared with wild-type U5 snRNA. A negative control using an empty pRS414 vector containing no U5 snRNA was also tested. U5 deletion cells containing U5 + ins displayed no growth defect when compared with U5 deletion strain containing the wild-type U5 snRNA gene in a pRS314 plasmid (Fig. 2). This lack of growth defect indicates that the 20 nucleotide insert in U5 + ins does not influence the function of the U5 snRNA.

The U5 snRNA mutants were tested for viability via plasmid shuffle at 16, 25, 30 and 37°C (Table 1). Of the U5 snRNA mutations in the 5′ side of IL1, Δ75–83 and 75–83 sub were both lethal and Δ78–81 and Δ79–80 were both viable, at all temperatures tested (Table 1). All four of the U5 Loop 1 mutants, Δ92–102, Δ92–95, Δ96–99 and Δ99–102, were lethal at all temperatures tested (Table 1). The U5 mutants Δ111–113 and Δ111–112 were lethal at 30 and 37°C, and displayed reduced growth at 25 and 16°C (Table 1). The U5 mutant Δ111 was lethal at 37°C, and displayed reduced growth at 25, 16 and 30°C (Table 1). Of all the U5 mutants tested in U5 + ins only 75–83 sub and Δ111 displayed different growth phenotypes when analysed within the wild-type U5 snRNA (Table S2).

**TABLE I:** Viability of U5 snRNA Mutants

	16°C	25°C	30°C	37°C
WT U5	+	+	+	+
U5 + ins	+	+	+	+
Δ75–83	−	−	−	−
Δ78–81	+	+	+	+
Δ79–80	+	+	+	+
75–83 sub	−	−	−	−
Δ111–113	+/−	+/−	−	−
Δ111–112	+/−	+/−	−	−
Δ111	+/−	+/−	+/−	−
Δ92–102	−	−	−	−
Δ92–95	−	−	−	−
Δ96–99	−	−	−	−
Δ99-102	−	−	−	−

Mutants constructed in the pROK4 plasmid (U5 + ins) which contains a 20 nucleotide insertion within stem 2 of the U5 snRNA. Wild-type growth (+), no growth (−), slow growth (+/−).

### U5 snRNA Requirements for Prp8, Snu114 and Brr2 Association

To investigate the influence of U5 snRNA mutations on the association of Prp8, Snu114 and Brr2 with the U5 snRNA, immunoprecipitations were performed to determine if specific U5 mutations caused a reduction in association. Yeast whole cell extracts were produced from strains containing TAP-tagged *BRR2* or *SNU114*, with both wild-type U5 snRNA (present in the genome) and mutant U5 snRNA present in the U5 + ins plasmid. Brr2 or Snu114 proteins were immunoprecipitated via the TAP tag. To investigate associations of Prp8 with U5 snRNA, extracts were produced from yeast strains containing wild-type and mutant U5 snRNA, but with no tagged protein present. Prp8 was immunoprecipitated with an anti-Prp8 antibody. All immunoprecipitations were carried out with a 150 mM salt concentration known to keep the Brr2/Snu114/Prp8 complex intact [Achsel et al., 1998; Zhang et al., 2009]. Following immunoprecipitation of Brr2-TAP, Snu114-TAP or Prp8, associated RNA was purified and subjected to primer extension using a primer specific for the U5 snRNA. Both wild-type and mutant U5 snRNAs were detected by the primer utilised. Mutant and wild-type U5 snRNA were identified by a size difference, because the 20 nucleotide insert present within the mutant U5 snRNAs produced larger primer extension products. A reduction in the amount of mutant U5 snRNA associated with the immunoprecipitated protein, compared with the level of U5 without mutation, would suggest an involvement of the mutated region in associations with that protein. The amount of associated U5 snRNA was detected and quantified by phosphorimaging. Primer extensions were also carried out with total RNA from each extract to illustrate that both the wild-type and U5 snRNA mutants were expressed in each extract and could be detected by primer extension. While variation was observed between extracts, this variation was observed for both wild-type U5 and U5 mutants within an extract indicating differences in the total RNA levels and not differences in expression of the U5 mutants. To control for any variability in immunoprecipitation, quantitation was normalised to the amount of endogenous U5 snRNA immunoprecipitated and experiments repeated as described in the Materials and Methods. Finally, western blotting was carried out on total protein from each extract, with an antibody to detect the protein to be immunoprecipitated and an antibody to detect glucose-6-phosphate dehydrogenase (G6PD) as a loading control, to confirm that U5 snRNA mutation did not significantly influence levels of Prp8, Snu114 or Brr2.

**Fig. 3:**
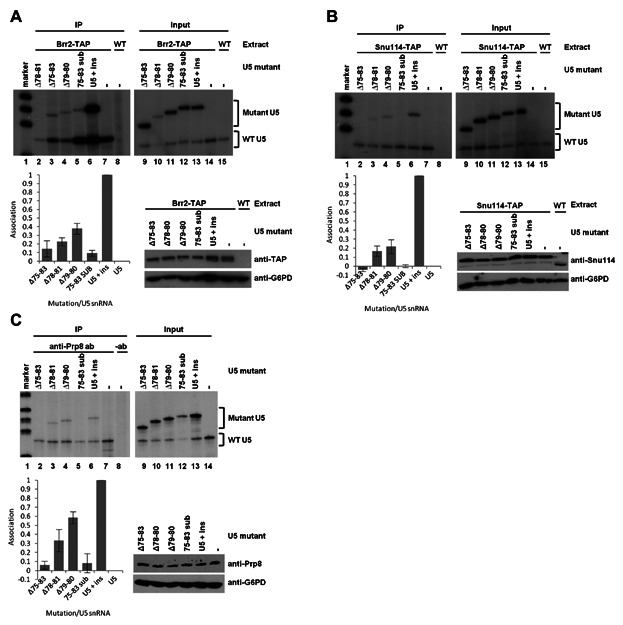
Influence of mutations in the 5′ side of U5 snRNA IL1 on Brr2 (A), Snu114 (B) or Prp8 (C) association with the U5 snRNA. Immunoprecipitation (IP) of Brr2-TAP or Snu114-TAP was carried out from extracts containing wild-type and mutant U5 snRNA. RNA associated with the immunoprecipitated protein was isolated and subjected to primer extension using a primer specific to the U5 snRNA. Negative controls using either untagged extract or using no Prp8 antibody were performed. Total RNA from each extract was also subjected to primer extension using a primer specific to U5 snRNA (Input). U5 snRNA mutants were constructed in a plasmid containing U5 snRNA with a 20 nucleotide insert (U5 + ins). Therefore, U5 snRNA mutants (Mutant U5) are detected as a larger product than wild-type U5 snRNA (WT U5). The experiments were repeated and quantified by phosphorimaging as described in the Materials and Methods Section. Graphical illustration is also shown of the amount of mutant U5 snRNA associated with Brr2, Snu114 or Prp8 in comparison with levels associated with U5 + ins without mutation. Western blotting was carried out on total protein from each extract to prove that the presence of U5 snRNA mutants does not influence levels of Brr2, Snu114 or Prp8 protein. Brr2 levels were detected using anti-TAP antibodies, Snu114 levels were detected using anti-Snu114 antibodies and Prp8 levels were detected using anti-Prp8 antibodies. Glucose-6-phosphate dehydrogenase (G6PD) was detected as a loading control using anti-G6PD antibodies.

To investigate how mutations in the 5′ side of U5 snRNA IL1 influenced associations of Prp8, Snu114 or Brr2 with the U5 snRNA, immunoprecipitations were carried out using extracts containing mutant and wild-type U5 snRNAs. All four mutations in the 5′ side of IL1 of U5 influenced the association of U5 snRNA with Prp8, Snu114 or Brr2 (Fig. 3). In the case of Brr2, the large deletion of 75–83 (Δ73–83) and the sequence substitution mutation (75–83 sub) had the largest effect, with the amount of U5 snRNA being immunoprecipitated reduced by 86% and 91%, respectively, compared with the U5 + ins without mutation (Fig. 3A). The smaller deletions, Δ78–81 and Δ79–80, also influenced associations of Brr2 with U5 snRNA. The levels of U5 mutants Δ78–81 and Δ79–80 immunoprecipitated with Brr2 were reduced by 77 and 62% respectively, compared with U5 + ins without mutation (Fig. 3A). Similarly, U5 Δ75–83 and U5 75–83 sub displayed the largest influence on Snu114 association with U5 snRNA, with the association of these mutant U5 snRNAs with Snu114 greatly reduced (Fig. 3B). The amount of U5 Δ78–81 and Δ79–80 associated with Snu114 was reduced to just 17 and 22% compared with U5 + ins without mutation (Fig. 3B). Continuing the trend observed with Brr2 and Snu114, the largest influence on associations between Prp8 and U5 snRNA was again with U5 Δ75–83 and U5 75–83 sub mutants. In both cases the amount of mutant U5 associated with Prp8p was reduced by 90% compared with U5 + ins without mutation (Fig. 3C). Deletion of U5 nucleotides 78–81 (Δ78–81) displayed a reduction of 67% in association with Prp8, compared with U5 + ins without mutation (Fig. 3C). However, the influence of U5 Δ79–80 was not as drastic, with U5 association reduced by only 41% compared with U5 + ins without mutation (Fig. 3C). In all cases, the presence of the U5 snRNA 5′ IL1 mutants did not influence the levels of Brr2, Snu114 or Prp8 protein (Fig. 3A–C). The general trend observed in these experiments is that deletion or substitution of U5 nucleotides 75–83 virtually abolishes association of Brr2, Snu114 or Prp8 with the U5 snRNA. Although deletion of U5 nucleotides 78–81 (Δ78–81) and 79–80 (Δ79–80) influences associations of Brr2, Snu114 or Prp8, the effect is not as drastic as with Δ75–83. As larger deletions of IL1 have a greater influence on protein association with the U5 the size of the 5′ side of U5 snRNA IL1 must be important. However, substituting U5 nucleotides 75–83 (75–83 sub) with the complement of the wild-type sequence nearly abolished the association of Prp8, Snu114 and Brr2 indicating that the sequence of the 5′ side of U5 snRNA IL1, not just size, is important for association of Prp8, Snu114 and Brr2. The association of Snu114 with the U5 snRNA appears to be most sensitive to mutations in the 5′ side of IL1, while the association of Prp8 with U5 snRNA is most tolerant to mutations in IL1.

**Fig. 4:**
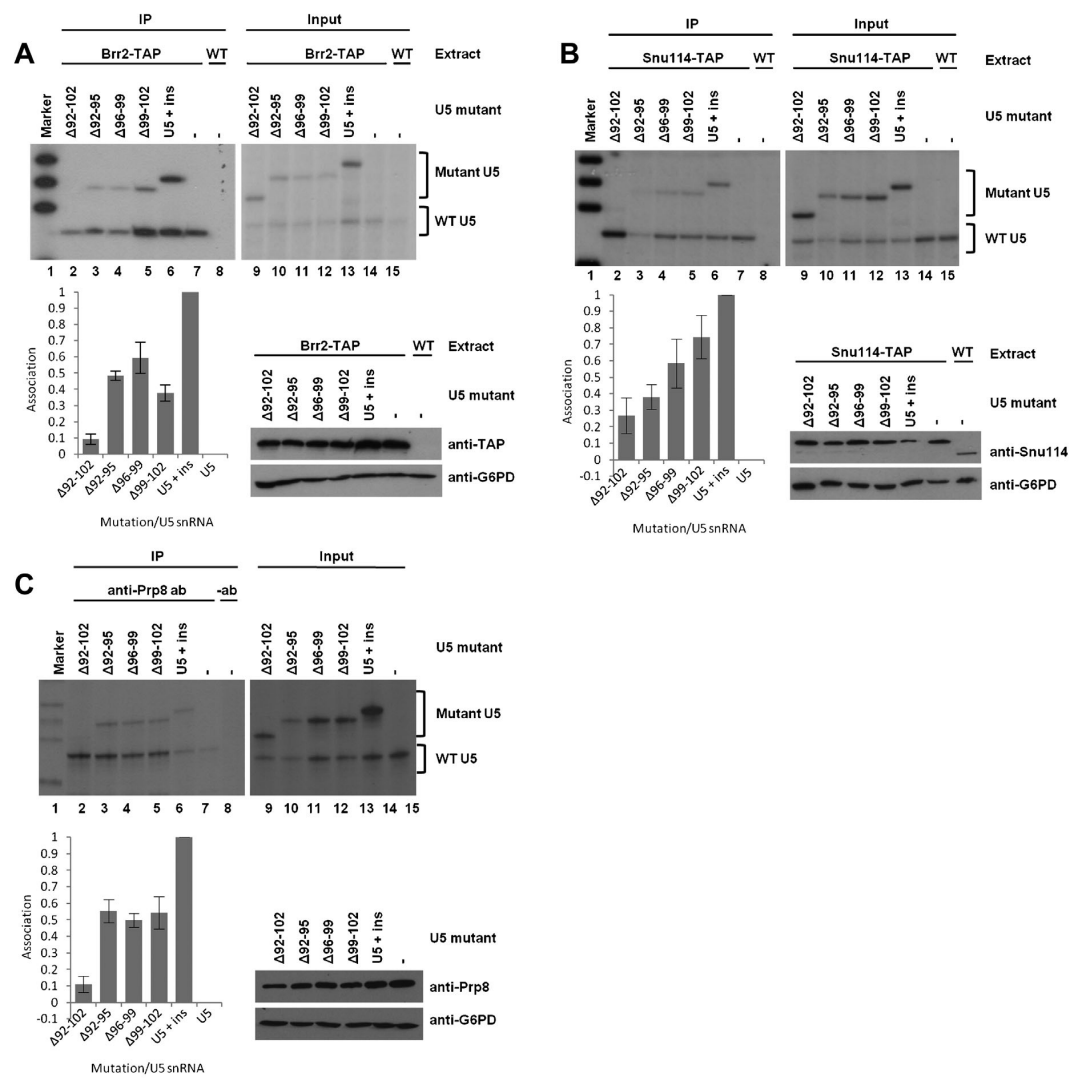
Influence of mutations in U5 snRNA loop 1 on Brr2 (A), Snu114 (B) or Prp8 (C) association with the U5 snRNA. Immunoprecipitation (IP) of Brr2-TAP or Snu114-TAP was carried out from extracts containing wild-type and mutant U5 snRNA. RNA associated with the immunoprecipitated protein was isolated and subjected to primer extension using a primer specific to the U5 snRNA. Negative controls using either untagged extract or using no Prp8 antibody were performed. Total RNA from each extract was also subjected to primer extension using a primer specific to U5 snRNA (Input). U5 snRNA mutants were constructed in a plasmid containing U5 snRNA with a 20 nucleotide insert (U5 + ins). Therefore, U5 snRNA mutants (Mutant U5) are detected as a larger product than wild-type U5 snRNA (WT U5). The experiments were repeated and quantified by phosphorimaging as described in the Materials and Methods Section. Graphical illustration is also shown of the amount of mutant U5 snRNA associated with Brr2, Snu114 or Prp8 in comparison with levels associated with U5 + ins without mutation. Western blotting was carried out on total protein from each extract to prove that the presence of U5 snRNA mutants does not influence levels of Brr2, Snu114 or Prp8 protein. Brr2 levels were detected using anti-TAP antibodies, Snu114 levels were detected using anti-Snu114 antibodies and Prp8 levels were detected using anti-Prp8 antibodies. Glucose-6-phosphate dehydrogenase (G6PD) was detected as a loading control using anti-G6PD antibodies.

To investigate how deletions in U5 snRNA loop 1 influenced association of Prp8, Snu114 or Brr2 with the U5 snRNA, immunoprecipitation and primer extension were carried out from extracts containing wild-type and mutant U5 snRNA. All the U5 snRNA loop 1 mutations influenced association of Brr2. The largest loop 1 deletion, U5 Δ92–102, displayed the most influence on Brr2 association, with levels of associated U5 Δ92–102 being reduced by 91% compared with U5 + ins without mutation (Fig. 4A). Of the three smaller four nucleotide deletions in loop 1, U5 Δ92–95 and U5 Δ99–102 displayed the largest influence, with levels of associated mutant U5 reduced by 52% and 62% respectively, compared with U5 + ins without mutation (Fig. 4A). Deletion of U5 nucleotides 96–99 (Δ96–99) had the least influence, with amounts of associated mutant U5 being reduced by 41% compared with U5 + ins without mutation (Fig. 4A). These results indicate that of the nucleotides present in U5 loop 1, nucleotides 92–102 are most important for the association of Brr2 with U5 snRNA.

All deletions in U5 snRNA loop 1 also influenced associations of Snu114 with the U5 snRNA. Deleting U5 snRNA loop 1 nucleotides 92–102 (Δ92–102) did not have as large an effect as with Brr2, as association of mutant U5 was reduced by 72% compared with U5 + ins without mutation (Fig. 4B). The three smaller U5 loop 1 deletions, Δ92–95, Δ96–99 and Δ99–102, reduced association of Snu114 with mutant U5 by 62%, 42% and 26%, respectively, compared with U5 + ins without mutation (Fig. 4B) suggesting that nucleotides 92–95 are more important than nucleotides 96–102 for the association of Snu114 with the U5 snRNA.

The largest U5 loop 1 deletion, Δ92–102, reduced Prp8 association with mutant U5 snRNA by 89% compared with U5 + ins without mutation (Fig. 4C). Each of the smaller deletions (Δ92–95, Δ96–99 and Δ92–102) had similar effects on Prp8 association with U5 snRNA, reducing association of Prp8 with mutant U5 snRNA by 45 and 50% compared with U5 + ins without mutation (Fig. 4C). The three smaller deletions all had equivalent effects suggesting that the size of U5 snRNA loop 1, not just sequence, is important for Prp8 association with U5 snRNA.

**Fig. 5:**
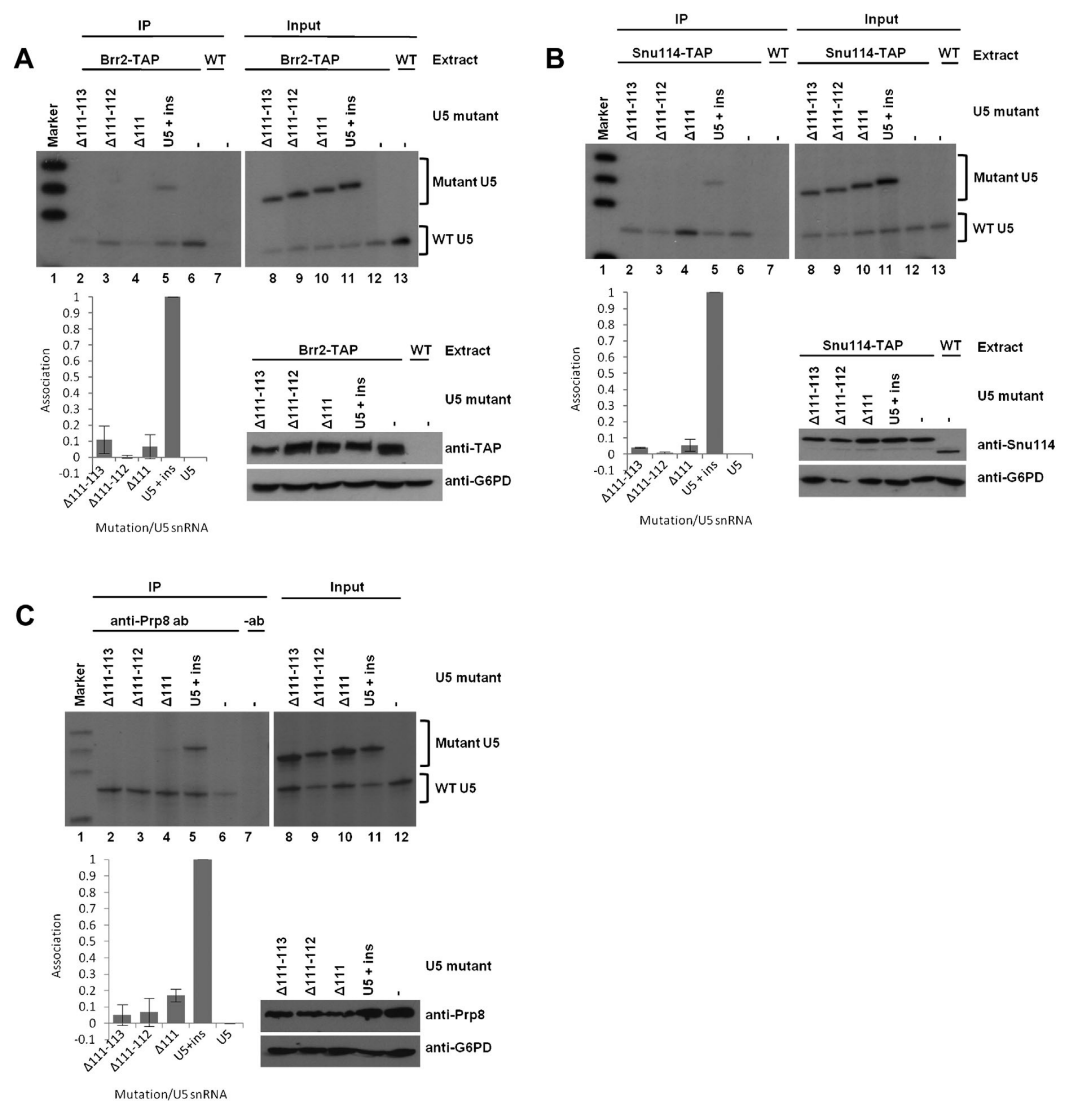
Influence of mutations in the 3′ side of U5 snRNA IL1 on Brr2 (A), Snu114 (B) or Prp8 (C) association with the U5 snRNA. Immunoprecipitation (IP) of Brr2-TAP or Snu114-TAP was carried out from extracts containing wild-type and mutant U5 snRNA. RNA associated with the immunoprecipitated protein was isolated and subjected to primer extension using a primer specific to the U5 snRNA. Negative controls using either untagged extract or using no Prp8 antibody were performed. Total RNA from each extract was also subjected to primer extension using a primer specific to U5 snRNA (Input). U5 snRNA mutants were constructed in a plasmid containing U5 snRNA with a 20 nucleotide insert (U5 + ins). Therefore, U5 snRNA mutants (Mutant U5) are detected as a larger product than wild-type U5 snRNA (WT U5). The experiments were repeated and quantified by phosphorimaging as described in the Materials and Methods Section. Graphical illustration is also shown of the amount of mutant U5 snRNA associated with Brr2, Snu114 or Prp8 in comparison with levels associated with U5 + ins without mutation. Western blotting was carried out on total protein from each extract to prove that the presence of U5 snRNA mutants does not influence levels of Brr2, Snu114 or Prp8 protein. Brr2 levels were detected using anti-TAP antibodies, Snu114 levels were detected using anti-Snu114 antibodies and Prp8 levels were detected using anti-Prp8 antibodies. Glucose-6-phosphate dehydrogenase (G6PD) was detected as a loading control using anti-G6PD antibodies.

To investigate the influence of mutation of the 3′ side of U5 snRNA IL1 on association of Prp8, Snu114 or Brr2 with U5 snRNA, immunoprecipitations from extracts containing wild-type and mutant U5 snRNA were performed, followed by primer extension. All deletions in the 3′ side of U5 snRNA IL1 reduced association of U5 with Brr2 by 89% or more, and with Snu114 by more than 94%, compared with U5 + ins without mutation (Fig. 5A,B). In the case of Prp8, deletion of U5 nucleotides 111–113 (Δ111–113) and 111–112 (Δ111–112) reduced associations by 95% and 93%, respectively, compared with U5 + ins without mutation (Fig. 5C). Deletion of U5 nucleotide 111 (Δ111) reduced association of mutant U5 with Prp8 by 83% compared with U5 + ins without mutation (Fig. 5C). These results demonstrate that associations of Prp8, Snu114 and Brr2 with the U5 snRNA are very sensitive to deletions in the 3′ side of U5 snRNA IL1.

### Genetic Interactions Between *brr2* and U5 snRNA Mutants

**Fig. 6:**
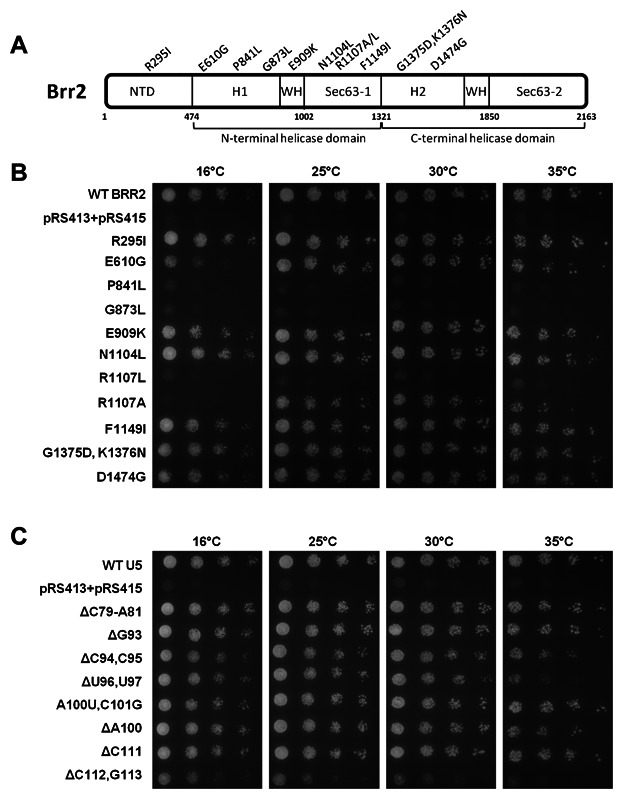
In vivo analysis of *brr2* and U5 snRNA mutants utilised for genetic screens. A: Four novel and seven previously published *brr2* mutants were constructed to investigate synthetic lethal interactions with the U5 snRNA (see Table 2 for details). The four novel *brr2* mutants contain substitutions of conserved amino acids in Brr2 (Fig. S1). The diagram illustrates the positions of the mutations in the Brr2 protein. The N-terminal domain (NTD), first and second RecA helicase-like domains (H1 and H2), the winged helix domains (WH) and the two Sec63 domains (Sec63-1 and Sec63-2) of Brr2 are also indicated on this diagram. B: The eleven *brr2* mutants were tested for viability via a plasmid shuffle assay, in a *BRR2*/U5 snRNA double deletion strain with both genes complemented with a pRS416-BRR2/U5 plasmid, in the presence of wild-type U5. One in five serial dilutions were spotted onto 5-FOA-containing plates. On each plate a positive control strain containing wild-type *BRR2* and U5, and a negative control strain containing pRS413 and pRS415 were also present. Spot plates were incubated at 16, 25, 30 and 37°C. C: The eight U5 snRNA mutants used in the genetic screen to investigate interactions with *BRR2*. The eight U5 snRNA mutants were tested for viability in the presence of wild-type *BRR2*. One in five serial dilutions were spotted onto 5-FOA-containing plates. On each plate a positive control strain containing wild-type *BRR2* and U5, and a negative control strain containing pRS413 and pRS415 were also present. Spotted plates were incubated at 16, 25, 30 and 37°C.

Both genetic and crosslinking studies have defined the interactions of Prp8 and Snu114 with the U5 snRNA [Dix et al., 1998; Grainger and Beggs, 2005; Frazer et al., 2009]. However, little is known about the physical and genetic interactions of Brr2 with the U5 snRNA, and the regions of Brr2 protein involved in these interactions. In addition, while genetic interactions with Brr2 have been identified with U5 snRNA loop 1 [Xu et al., 1998], no information is available on interactions with U5 IL1 which we have found to be important for association of Brr2 with the U5 snRNA. To investigate genetic interactions between *BRR2* and the U5 snRNA, seven published and four novel *brr2* mutants were constructed (Table 2, Fig. 6A). The four novel *brr2* mutants were chosen as the four amino acids changed are highly conserved in Brr2 from yeast to humans (Fig. S1). All *brr2* mutants constructed were tested for viability via plasmid shuffle using a haploid *BRR2*/*SNR7* (U5 snRNA) double deletion strain with the gene deletions complemented by wild-type *BRR2* and U5 snRNA together on a *CEN*/*URA3* plasmid. The *BRR2*/U5 snRNA deletion strain was co-transformed with a *brr2* mutant and wild-type U5 snRNA. Transformants were transferred to 5-FOA-containing media and tested for viability at 16, 25, 30 and 35°C (Fig. 6B). The novel *brr2* mutants containing mutations in the first helicase-like domain (H1), Brr2-P841L and Brr2-G873L, and mutation R1107L were all lethal at the temperatures tested (Fig. 6B). The novel *brr2* N-terminal mutant, Brr2-R295I, mutant E909K in the winged helix domain (WH) and two *brr2* alleles containing mutations in the first Sec63 domain (Sec63-1), Brr2-N1104L and Brr2-F1149I, were viable at all temperatures tested (Fig. 6B). Both *brr2* mutants containing mutations in the second helicase-like domain (H2), G1375D/K1376N and D1474G, were also viable at all temperatures tested (Fig. 6B). The H1 mutant, Brr2-E610G was sick at 16°C and viable at all other temperatures tested (Fig. 6B). The Sec63-1 mutant, Brr2-R1107A, was not viable at 16°C, but viable at 25, 30 and 35°C (Fig. 6B). The lethal phenotype of the two novel H1 mutants, and the viability of the two H2 mutants supports the hypothesis that it is the first helicase domain that functions in the essential process of U4/U6 unwinding [Kim and Rossi, 1999].

**TABLE II:** Eleven *brr2* Mutants Constructed for Synthetic Lethal Screens with U5 snRNA Mutants

*brr2* mutant	Refs.	Viability temperatures^a^
R295I	This study	16, 25, 30, 35
E610G (*brr2-1*)	Raghunathan and Guthrie [1998]	16, 25, 30, 35
P841L	This study	Inviable
G873L	This study	Inviable
E909K (*slt22-1*)	Xu et al. [1996]	16, 25, 30, 35
N1104L	Zhao et al. [2009]	16, 25, 30, 35
R1107A	Small et al. [2006]	25, 30, 35
R1107L	Zhao et al. [2009]	Inviable
F1149I	This study	16, 25, 30, 35
G1375D, K1376N	Kim and Rossi [1999]	16, 25, 30, 35
D1474G	Kim and Rossi [1999]	16, 25, 30, 35

a Viability temperatures in °C.

To investigate genetic interactions between *BRR2* and U5 snRNA, a genetic screen was carried out using the viable *brr2* mutants and a set of viable U5 snRNA mutants. The U5 mutants chosen for use in the screen were U5 ΔC79-A81, containing a deletion in the 5′ side of U5 snRNA IL1, two mutants containing deletions in the 3′ side of U5 snRNA IL1, ΔC111 and ΔC112G113, and several mutations in U5 loop 1. The U5 loop 1 mutants were ΔG93, ΔC94C95, ΔU96U97 and ΔA100 and A100UC101G. U5 snRNA mutations in the IL1 and loop 1 of U5 snRNA were selected for use in this screen as Snu114 is known to crosslink to IL1, and Prp8p is known to crosslink to both IL1 and loop 1 [Dix et al., 1998]. Snu114 has also been shown to have genetic interactions with loop 1 and IL1 of U5 snRNA [Frazer et al., 2009].

Prior to use in the genetic screen, the U5 mutants were tested for viability without any *brr2* mutants present, in combination with wild-type *BRR2*, at 16, 25, 30 and 35°C (Fig. 6C). Of the U5 snRNA loop 1 mutants used in the screen, ΔC79-A81, ΔG93, ΔC94C95, A100UC101G, ΔA100 and ΔC111 were viable at all temperatures tested. The U5 ΔU96U97 was viable at 16, 25 and 30°C, but sick at 35°C. The U5 snRNA mutant ΔC112G113 was sick at 16, 25 and 30°C, and lethal at 35°C.

**Fig. 7:**
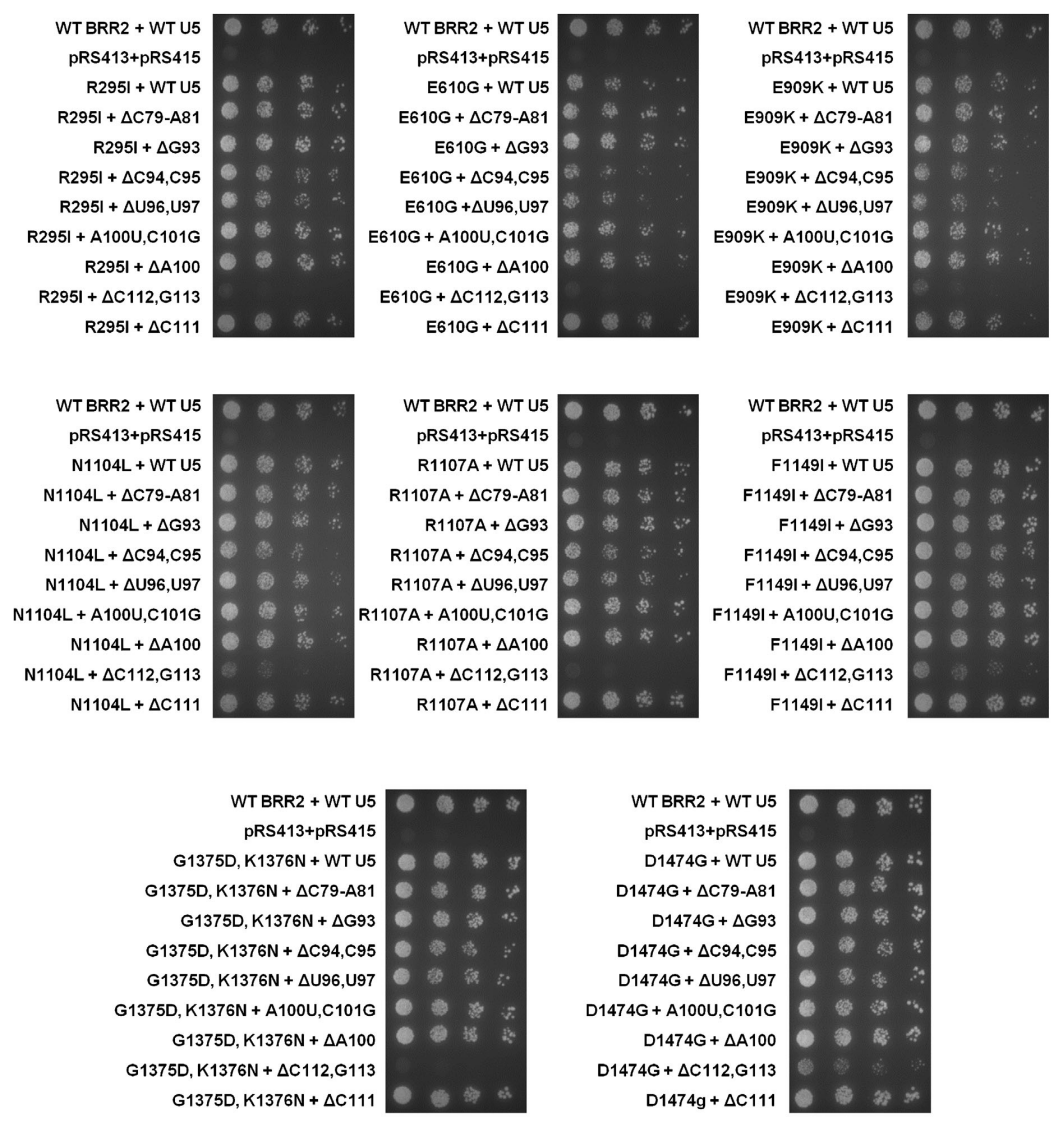
Genetic interactions between the *brr2* mutants and U5 snRNA mutants. Eight viable *brr2* mutants were tested for genetic interactions with eight viable U5 snRNA mutants by plasmid shuffle. The interaction assays were performed in a *BRR2*/U5 double deletion strain, carrying the wild-type genes on a single *URA3* plasmid. One in five serial dilutions were spotted onto 5-FOA-containing plates and incubated at 25°C for 3 days.

A genetic screen was carried out, testing every combination of viable *brr2* mutant and U5 snRNA mutants via plasmid shuffle at 25°C (Fig. 7). Of the 64 mutant combinations tested, five genetic interactions were found (Fig. 7). These genetic interactions were only found with one mutation in U5 IL1, ΔC112G113. Brr2 mutants R295I, E610G, R1107A and G1375D, K1376N were lethal when combined with U5 ΔC112G113, whereas E909K was very sick combined with U5 ΔC112G113 when compared to the growth of U5 ΔC112G113 with wild-type Brr2 observed at 25°C in Figure 6C. These results would suggest that the 3′ side of U5 snRNA IL1 is essential for Brr2 function in vivo.

**Fig. 8:**
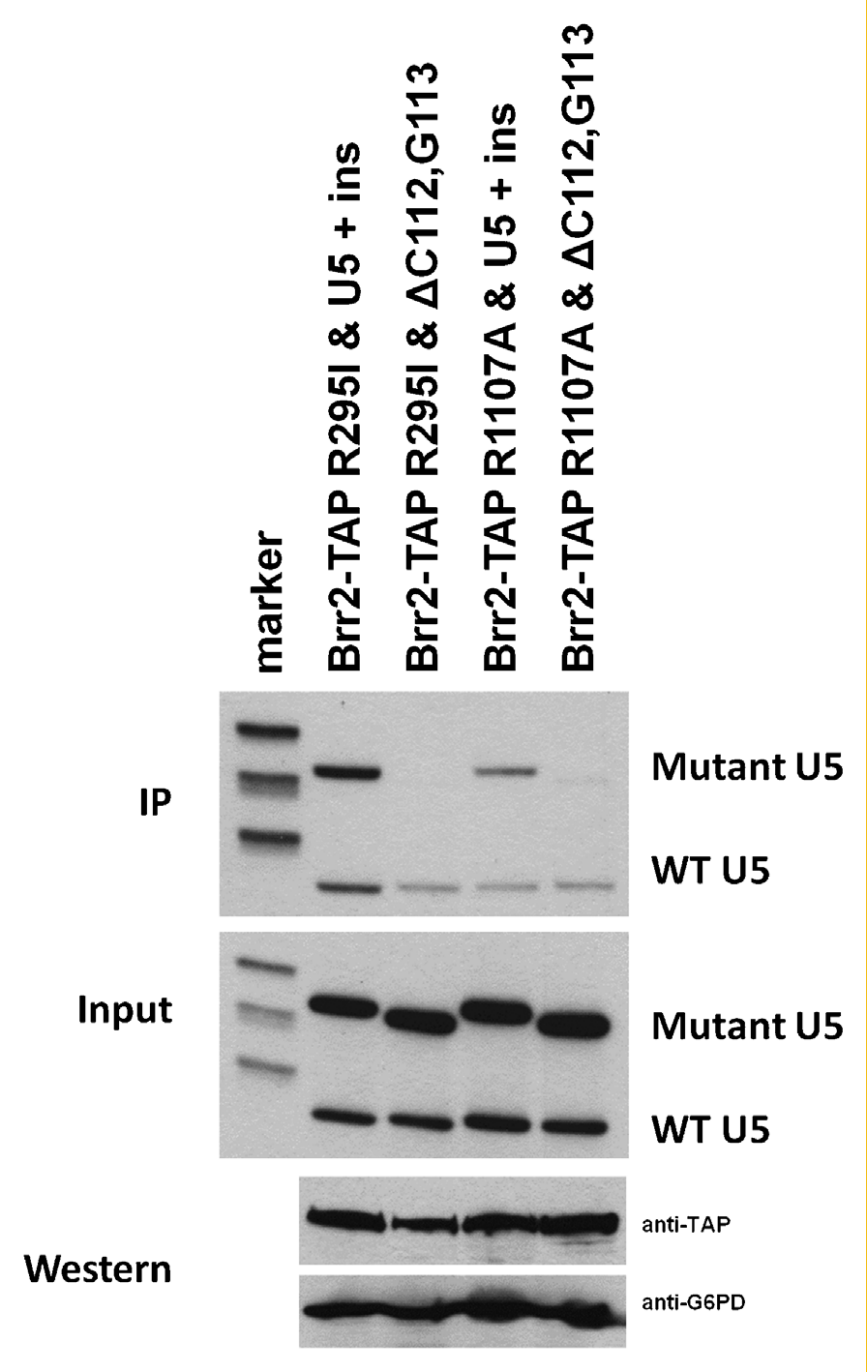
Genetic interactions reflect reduced association between Brr2 and U5. Immunoprecipitation (IP) of Brr2-TAP was carried out from extracts containing wild-type and mutant U5 snRNA. RNA associated with the immunoprecipitated protein was isolated and subjected to primer extension using a primer specific to the U5 snRNA. Total RNA from each extract was also subjected to primer extension using a primer specific to U5 snRNA (Input). U5 snRNA mutants were constructed in a plasmid containing U5 snRNA with a 20 nucleotide insert (U5 + ins). Therefore, U5 snRNA mutants (Mutant U5) are detected as a larger product than wild-type U5 snRNA (WT U5). Western blotting with anti-TAP antibodies was carried out on total protein from each extract to prove that the presence of U5 snRNA mutants does not influence levels of Brr2 protein. Glucose-6-phosphate dehydrogenase (G6PD) was detected as a loading control using anti-G6PD antibodies.

The genetic interactions found between the Brr2 mutants and the U5 IL1 may not necessarily reflect direct binding or physical interactions between Brr2 and the U5 snRNA. Therefore, to determine whether the genetic interactions observed reflect a change in association of Brr2 with the U5 snRNA, immunoprecipitation was carried out with selected Brr2 mutants found to display genetic interactions with the U5 IL1 mutation ΔC112G113. A plasmid copy of TAP-tagged Brr2 with the R295I or R1107A mutation was transformed into a yeast strain together with the plasmid U5 + ins or U5 + ins containing the ΔC112G113 mutation. Primer extension of total input RNA isolated from extracts produced from these strains revealed that the expression levels of wild-type and mutant U5 was consistent between extracts (Fig. 8). Western blotting was carried out on total protein from each extract to confirm that U5 snRNA mutation did not significantly influence levels of Brr2-TAP compared to the G6PD loading control (Fig. 8). Extracts from these strains were then subjected to immunoprecipitation of the TAP-tagged Brr2 and associated U5 snRNA was analysed by primer extension (Fig. 8). A significantly reduced association of the ΔC112G113 U5 mutant was observed with the Brr2 R295I and R1107A, whereas both the Brr2 R295I and R1107A mutants still associated with the wild-type and U5 + ins U5 snRNA. Therefore, it appears that the synthetic lethal interactions observed with the Brr2 R295I or R1107A mutant combined with the U5 ΔC112G113 mutation reflects a reduced association between Brr2 and the U5 snRNA IL1.

## DISCUSSION

We have investigated the requirements for association of the U5 snRNP proteins Prp8, Snu114 and Brr2 with the U5 snRNA. The U5 snRNA IL1 and loop 1 are important for association of Prp8, Snu114 and Brr2 with the U5 snRNA. Mutations in U5 IL1 influenced the association of Prp8, Snu114 and Brr2 the most, supporting the hypothesis that U5 IL1 forms a platform for protein binding to the U5 snRNA.

U5 snRNA mutants were constructed and tested for viability as the sole source of U5 snRNA in vivo within a U5 snRNA gene containing a 20 nucleotide insertion in stem 2. It was surprising that deletion of nucleotides 78–81 (Δ78–81) and 79–80 (Δ79–80), from the 5′ side of U5 snRNA IL1 did not result in a growth phenotype at any temperature tested as the C79G80 dinucleotide is invariant in all U5 species [Frank et al., 1994]. This lack of growth phenotype indicating that the 5′ side of U5 snRNA IL1 is resilient to deletions and the invariant C79G80 must be dispensable. It is known that position C79 crosslinks with both Snu114 and Prp8 [Dix et al., 1998]. It is possible that C79G80 are both involved in protein interactions, but are not the only site of protein interaction. Positions C79G80 have also been proposed to form a base-pairing interaction with nucleotides on the 3′ side of IL1 [Mougin et al., 2002]. Because deletion of C79G80 does not display a lethal phenotype the function of this base-pairing interaction may not be essential for U5 snRNA function.

All deletions in U5 snRNA loop 1 resulted in a lethal phenotype because loop 1 is essential for the alignment of exons during the second step of splicing [Newman and Norman, [Bibr b29],[Bibr b30]; O'Keefe et al., 1996; O'Keefe and Newman, 1998]. Mutation in U5 snRNA that reduces the size of loop 1 influences the stability of Prp8, thus affecting U5 snRNP and tri-snRNP assembly [Kershaw et al., 2009]. However, because the wild-type U5 snRNA was present, in addition to the U5 snRNA mutations we investigated, Prp8 would remain stable. This stability was confirmed by western analysis of Prp8 that revealed no significant change in Prp8 levels in the presence of any U5 snRNA mutants.

Deletion of nucleotide C111 of U5 snRNA resulted in a sick phenotype at 30°C (Table 1). This ΔC111 mutation was viable at 30°C [Frazer et al., 2009] and at other temperatures when tested here without the 20 nucleotide insertion (Table S2). The 75–83 sub mutation was lethal at all temperatures when tested with the 20 nucleotide insertion whereas 75–83 sub was either sick or viable without the 20 nucleotide insertion. It is therefore apparent that within the context of these two mutations the 20 nucleotide insertion used to distinguish wild-type from mutant U5 may be influencing U5 function.

The U5 snRNP is found as free U5 snRNP, as part of the U4/U6.U5 tri-snRNP and as part of the assembled and active spliceosome. As we have investigated Prp8, Snu114 and Brr2 association with the U5 snRNA in whole cell extracts it is the assembly of the free U5 snRNP which the U5 snRNA mutations will primarily affect. All deletions made in the 5′ side of U5 snRNA IL1 influenced associations of Prp8, Snu114 and Brr2 with U5 snRNA. Of the three mutants containing deletions in the 5′ side of U5 IL1, the largest deletion (Δ75–83) had the largest influence on association of each protein. The four nucleotide deletion (Δ78–81) also influenced associations of Prp8, Snu114 and Brr2 with U5 snRNA, while the two nucleotide deletion (Δ79–80) had the smallest influence. This general trend, where influence on protein association is proportional to the size of deletion suggested that the size of the 5′ side of U5 IL1 was important for Prp8, Snu114 and Brr2 association, as indicated by the high conservation of IL1 size between U5 snRNAs from different species [Frank et al., 1994].

Although the general trends of how the mutations in the 5′ side of U5 IL1 influenced association of Prp8, Snu114 and Brr2 were the same for each protein, the association of Snu114 with U5 was particularly sensitive. Of the three proteins investigated, the association of Prp8 was least influenced by mutations in the 5′ side of U5 snRNA IL1. The large deletion (Δ75–83) and the sequence substitution (75–85 sub) practically abolished the association of the mutant U5 with Snu114, whereas Brr2 and Prp8 still displayed some association. Where protein association is still seen with U5 mutants, it is possible that only one of the three proteins, Prp8, Snu114 or Brr2, is interacting with U5, and the other proteins are associating with U5 indirectly, via the other protein(s). The association of Snu114 with U5 was practically abolished with U5 Δ75–83 and 75–83 sub, but Prp8 and Brr2 still displayed some association with the mutant U5. It is not surprising that Prp8 still displayed some association with the mutant U5 snRNAs, because Prp8 crosslinks to five different positions in U5 [Dix et al., 1998]. Not only is the interaction between Prp8 and the U5 snRNA direct, but it is also extensive, so even in the absence of the 5′ side of U5 IL1, Prp8 could still associate weakly with other regions of U5. Brr2 still associates with U5 Δ75–83 and 75–83 sub, so it is possible that Brr2 is binding U5 indirectly, through known protein-protein interactions with the C-terminus of Prp8 [van Nues and Beggs, 2001; Liu et al., 2006]. However, because the Snu114 association with U5 Δ75–83 and 75–83 sub is so low, protein-protein interactions between Snu114 and Prp8 under the conditions used here for immunoprecipitation are not sufficient for Snu114 association with these U5 mutants. Association of Snu114 with the U5 snRNA may require a direct interaction with the 5′ side of U5 IL1 which is supported by Snu114 crosslinking to the 5′ side of U5 snRNA IL1 and by synthetic sick interactions identified between Snu114 and IL1 of U5 [Dix et al., 1998; Frazer et al., 2009].

All U5 loop 1 deletions influenced association of Prp8, Snu114 and Brr2 with U5. The largest deletion in U5 loop 1, Δ92–102, had the greatest impact on association of Prp8, Snu114 and Brr2 with U5. The influence of Δ92–102 on association of Snu114 was not as large as that seen with Brr2 and Prp8, suggesting that the association of Snu114 is less sensitive to deletions in loop 1 of U5. The decreased sensitivity of Snu114 association with a major deletion in U5 loop 1 suggests that Snu114 also interacts with another region of U5 snRNA. This region is likely the 5′ side of IL1, which would allow association of Snu114 with U5 in the absence of U5 loop 1. The influence of the U5 loop 1 92–102 deletion on Brr2 and Prp8 association with U5 were very similar indicating that Brr2 may be interacting with U5 via Prp8, which crosslinks extensively to U5 [Dix et al., 1998]. The three smaller, four nucleotide, deletions (Δ92–95, Δ96–99 and Δ99–102), did not have the same influence on associations of Prp8, Snu114 and Brr2 with U5 snRNA. In the case of Brr2, U5 Δ99–102 had the largest influence while U5 Δ96–99 had the least influence. U5 Δ99–102 having more of an influence on Brr2 associations than U5 Δ92–95 or Δ96–99, would suggest that nucleotides 99–102 are most important for the association of Brr2 with U5 snRNA. Therefore, both Prp8 and U5 nucleotides 99–102 may form the necessary structure required for Brr2 association with the U5 snRNA.

Of the smaller deletions in U5 loop 1, Δ92–95 had the largest influence on the association of Snu114 with U5, and Δ99–102 had the smallest influence, with a reduction in association of only around 25% compared with U5 with no mutation. U5 Δ96–99 had an intermediate effect on associations of Snu114 with U5 snRNA. This pattern of influence would indicate that nucleotides in the 5′ side of U5 loop 1 are more important for the association of U5 snRNA with Snu114 than nucleotides in the 3′ half of loop 1. Together with the observation of Snu114 crosslinking to the 5′ side of IL1, these data suggest that Snu114 contacts U5 on the 5′ side of stem/loop 1 [Dix et al., 1998].

All U5 snRNA mutants containing four nucleotide deletions in loop 1 (Δ92–95, Δ96–99 and Δ99–102) reduced association of Prp8 by 45–50%. Because each of the deletions of four nucleotides had a similar influence on association of Prp8 with U5 snRNA, it would suggest that the size of U5 loop 1 is important for the association of Prp8. Prp8 has been shown to crosslink to position U97 in loop 1 from yeast and U40–U43 (equivalent to yeast U96–U99) in loop 1 from humans [Dix et al., 1998; Urlaub et al., 2000]. Prp8 is still associating with U5 snRNA in the absence of some nucleotides in loop 1 demonstrates that these nucleotides are only moderately important for the association of Prp8.

All deletions in the 3′ side of U5 IL1 resulted in the largest reduction in association of Prp8, Snu114 and Brr2 with U5 although these deletions had slightly less influence on associations of U5 with Prp8, than with Brr2 and Snu114. With all deletions in the 3′ side of U5 IL1 having such a large impact, it is possible that U5 IL1 acts as a protein docking site within the U5 snRNP, tethering the Prp8, Snu114 and Brr2 complex to U5. Even a single nucleotide deletion in the 3′ side of U5 IL1 had drastic influence on the associations of Prp8, Snu114 and Brr2 with the U5 snRNA. The importance of the 3′ side of U5 IL1 is emphasised by specific genetic interactions of the Brr2 observed in this study. Genetic interactions have also been found with the N- and C-termini of Snu114 with the 3′ side of IL1 [Frazer et al., 2009]. Finally, Prp8 crosslinks to position C112 in the 3′ side of IL1 [Dix et al., 1998]. Taken together, the immunoprecipitation, genetic and crosslinking data would suggest a model in which the N- and C-termini of Snu114, Brr2, and an undefined region of Prp8, associate with the 3′ side of IL1 of U5.

Prp8, Snu114 and Brr2 have been found to form a salt resistant complex in the absence of the U5 snRNA and some *snu114* mutants prevent Prp8/Snu114 interaction resulting in decreased U5 snRNP [Achsel et al., 1998; Brenner and Guthrie, 2006]. These results, combined with our extensive analysis of Prp8, Snu114 and Brr2 association with mutant U5 snRNAs shown here revealing no major differences, suggest that these three proteins almost certainly assemble with the U5 snRNA mostly as a complex. Whether this complex of Prp8, Snu114 and Brr2 interacts with U5 through just one, two or all of the proteins remains to be determined.

Viable mutations in *brr2* were combined with viable mutations in U5 snRNA loop 1 and IL1 to search for genetic interactions between these factors. Interestingly, synthetic lethal and sick interactions were only found between *brr2* mutations and one mutation in U5 IL1 (ΔC112G113) supporting the importance of IL1 in U5 snRNP function. Genetic interaction does not necessarily reflect disruption of a direct interaction between Brr2 and the U5 IL1. However, analysis of two synthetic lethal interactions by immunoprecipitation revealed that the mutations R295I and R1107A displayed dramatically reduced association with the U5 snRNA ΔC112G113 mutation. Because Brr2 forms a complex with both Prp8 and Snu114 under the conditions we are using for the immunoprecipitation, the reduced association of Brr2 mutants with U5 ΔC112G113 may not be direct and may result from disruption of Prp8 and/or Snu114 interaction with IL1. Our results, therefore, suggest that the integrity of U5 IL1 is directly or indirectly important for Brr2 association with the U5 snRNA and Brr2 function. It is possible interaction of Prp8 and/or Snu114 with U5 IL1 may be required for the regulation of Brr2 unwinding activity by Prp8 and Snu114. Our proposed role of U5 IL1 as a platform for Prp8, Snu114 and Brr2 association with the U5 snRNA, suggests a structural role of IL1 in U5 snRNP formation and also a possible functional role in regulating Brr2 activity.
