# Supplementary material for: The U5 snRNA Internal Loop 1 Is a Platform for Brr2, Snu114 and Prp8 Protein Binding During U5 snRNP Assembly
Source: J Cell Biochem. 2013 Jul 16;114(12):2770–84. doi: 10.1002/jcb.24625 (PMC4065371; doi:10.1002/jcb.24625)
Supplement: Supplementary file 1 [file jcb0114-2770-SD1.pdf]

Supplemental Material to:

Nancollis V, Ruckshanthi JPD, Novak Frazer L, O'Keefe RT

The U5 snRNA internal loop 1 is a platform for Brr2, Snu114 and Prp8 protein binding during U5 snRNP assembly

Included: Figure S1, Table S1 and Table S2

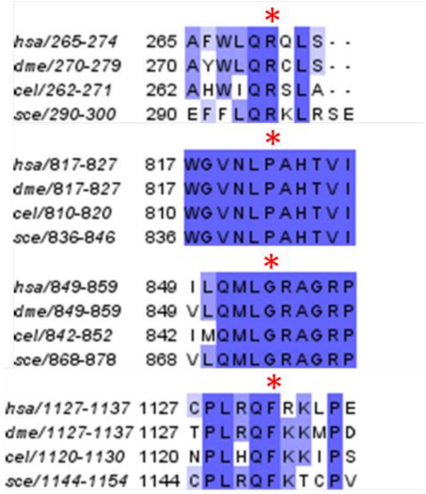

Figure S1. Conserved residues of Brr2p chosen for mutagenesis.

An alignment of the amino acid sequence of regions of Brr2p from *Homo sapiens* (hsa), *Drosophila melanogaster* (dme), *Caenorhabditis elegans* (cel) and *Saccharomyces cerevisiae* (sce). The figure illustrates the conserved nature of amino acids R295, P841, G873 and F1149 that were mutated to create novel Brr2p mutants.

Table S1. Primers used for primer extension (RT) and oligomutagenesis

Primer name	Sequence
U1 RT 136	GACCAAGGAGTTTGCATCAATGAC
U2 RT all	GCCAAAAAATGTGTATTGTAAC
U4 RT all	GGTATTCCAAAAATTCCCTACATAGTC
U5 RT	AAAAATATGGCAGG CCTACAGTAACGG
U6 RT all	TCATCCTTATGCAGGG
U5 Δ75-83	GGCAAGAACCATAAGTTCTATAGGC
U5 Δ78-81	GCAAGAACCATGTTATAAGTTCTATAGG
U5 Δ79-80	CAAGAACCATGTTTTATAAGTTCTATAG
U5 75-83 sub	GGCAAGAACCATCAAGCAATAAAGTTCTATAGG
U5 Δ92-102	CCGGATGGTTCTAGAACCATGTTCG
U5 Δ92-95	GGTTCTGGTAAAAAGAACCATGTTGC
U5 Δ96-99	GATGGTTCTGGTGGCAAGAACCAT
U5 Δ99-120	CCGGATGGTTCTAAAGGCAAGAACC
U5 Δ111-113	GCATACTTCTACAACACCATGGTTCTGGTAAAAGG
U5 Δ111-112	GCATACTTCTACAACACCCATGGTTCTGGTAAAAGG
U5 Δ111	GCATACTTCTACAACACCCGATGGTTCTGGTAAAAGG
Brr2-R295I	TGACCGAAGTTTAATCTGCAAAAAAAAATTC
Brr2-E610G	TATGTCCCATTTCCCTGGCGTAGACAC
Brr2-P841L	CACTGTATGAGCCAATAGATTAACACC
Brr2-G873L	TCTGCCTGCTCTCAATAACATTTGAAG
Brr2-E610G	TATGTCCCATTTCCCTGGCGTAGACAC
Brr2-E909K	AACAAATTGCGATTTTATTGGTAATTG
Brr2-N1104L	CAATAACCTACCAGCCAATTGGTGAATAAACAC
Brr2-R1107A	AGCACGCAATAACGCACCAGCATTTTG
Brr2-R1107L	CATAGCACGCAATAACAAACCAGCATTTTGGTG
Brr2-F1149I	GTCCTTTAAGACAGATTAAAACATGCCCTGTC
Brr2-G1375D,K1376N	AGCCATAGCTGTATTATCTGTGCCCTTTCC
Brr2-D1474G	CTCATGAGCGTCACCATATATCATTAA
Brr2FG	ACAATTGGGCTTGAACTTTTCG
Brr2BG	AAAACGACTTCTTATCTTTCG

Table S2. Viability of U5 snRNA mutants

	16°C	RT	30°C	37°C
WT U5	+	+	+	+
Δ75-83	-	-	-	-
Δ78-81	+	+	+	+
Δ79-80	+	+	+	+
75-83 sub	+/-	+/-	+	+
Δ111-113	+/-	+/-	-	-
Δ111-112	+/-	+/-	-	-
Δ111	+	+	+	+
Δ92-102	-	-	-	-
Δ92-95	-	-	-	-
Δ96-99	-	-	-	-
Δ99-102	-	-	-	-

Mutants constructed in the wild-type U5 snRNA plasmid (m571). Room temperature (RT), wild-type growth (+), no growth (-), slow growth (+/-). Yellow shaded growth results indicate those that are different to the same mutation in the pROK4 plasmid (U5 + ins).
